# Health-Promoting Activities and Associated Mechanisms of Polygonati Rhizoma Polysaccharides

**DOI:** 10.3390/molecules28031350

**Published:** 2023-01-31

**Authors:** Shuzhen Wang, Feng He, Hongmei Wu, Fu Xiang, Hongyan Zheng, Wei Wu, Shiming Li

**Affiliations:** 1 College of Biology and Agricultural Resources, Huanggang Normal University, Huanggang 438000, China; 2 Department of Library, Huanggang Normal University, Huanggang 438000, China; 3 Misabel Group Inc., Shijiazhuang 051134, China; 4 Department of Food Science, Rutgers University, New Brunswick, NJ 08901, USA

**Keywords:** Polygonati Rhizoma, homology of medicine and food, polysaccharides, health-promoting property, synergy mechanism

## Abstract

Polygonati Rhizoma, a typical homology of medicine and food, possesses remarkable anti-fatigue, anti-aging, metabolic regulatory, immunomodulatory, anti-inflammatory, neuroprotective, anti-diabetes, and anti-cancer effects. Among bioactive phytochemicals in Polygonati Rhizoma, polysaccharides play important roles in the health-promoting activities through the mechanisms mentioned above and potential synergistic effects with other bioactives. In this review, we briefly introduce the updated biosynthesis of polysaccharides, the purification method, the structure characterization, and food applications, and discuss in detail the biological activities of Polygonati Rhizoma polysaccharides and associated mechanisms, aiming at broadening the usage of Polygonati Rhizoma as functional food and medicine.

## 1. Introduction

Well-recorded in the Commission of Chinese Medicine Dictionary and Commission of Chinese Pharmacopoeia, Polygonati Rhizoma (“Huangjing” in Chinese) possesses remarkable health-promoting activities, including replenishing Qi, nourishing Yin, moistening lungs, fortifying spleens, tonifying kidneys, replenishing energy, and strengthening immunity [1,2]. Widely used in traditional Chinese medicine, Polygonati Rhizoma is used in treating fevers of influenza, mastitis, dizziness, coughs, fatigue, feebleness, diabetes mellitus, sexual dysfunction, indigestion, inappetence, backache, keen pain, and lung trouble [3,4,5]. As a typical homology of medicine and food, annual demand for Polygonati Rhizoma continues to rise [6].

In particular, Polygonati Rhizoma is prepared from rhizomes of *Polygonatum sibiricum* Redoute, *P. kingianum* Coll.et Hemsl and *P. cyrtonema* Hua [7,8,9]. Among the three raw traditional herbs, *P. cyrtonema* is optimal both in quality and yield, endemic to China (central and eastern) and widely cultivated under forest [10]. Meanwhile, *P. sibiricum* is mainly distributed in northern China, North Korea, and Mongolia [11]. Furthermore, *P. kingianum*, also native to southern China, is mainly distributed in the Yunnan, Sichuan, Guizhou and Guangxi provinces, among others [12]. Up to now, 365 kinds of commercial food products and more than 500 patents associated with Polygonati Rhizome are available [13].

**Figure 1:**
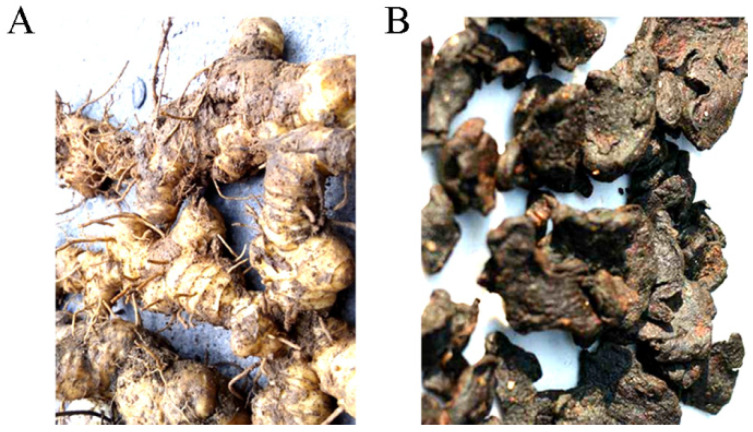
The traditional Chinese medicine Polygonati Rhizoma: (**A**) rhizome; (**B**) Polygonati Rhizoma.

Mature rhizomes of *P. sibiricum*, *P. kingianum*, and *P. cyrtonema* are yellow and branched, and need to be processed through the strategy of nine cycles of steaming and drying [14]. The processed Polygonati Rhizomes with enhanced function are black, soft, and sweet, without causing throat irritation (Figure 1) [15]. Repeated steaming treatment could influence physicochemical properties and bioactivities of Polygonati Rhizoma active substances, as molecular weights, UV absorption, and antioxidant activity of polysaccharides were significantly increased [16]. If considering total saccharides, fructose (Fru) and phospholipids as the main quality indicators, four cycles of steaming-drying should be the ideal method to obtain better taste, flavor and functionality [14]. If considering antioxidant and immunopotentiation activities, two cycles of continuous steaming-drying would be the best choice [17].

Polygonati Rhizoma contains many bioactive substances, such as purine nucleoside [18], carbohydrates [19,20], bioflavonoids [21], alkaloids [22], saponins [23,24], lignins, amino acids, peptide, anthraquinone, cardiac glycosides, vitamins, and various acids [25,26]. Polysaccharides are major bioactive components and contributors to the sweet taste of Polygonati Rhizoma which mainly exert metabolic regulation, immunomodulatory, anti-fatigue, anti-aging, antiviral, anti-inflammatory, antioxidant, antiatherosclerotic, liver protection, hypolipidemic, anti-osteoporosis, anti-cancer, anti-diabetic, and anti-atherosclerotic activities [27,28,29]. Polysaccharides also serve as evaluation markers in quality control of Polygonati Rhizoma and “Yuzhu” (*P. odoratum*) [30,31]. Polygonati Rhizoma has often been used as an ingredient or supplement in the food industry due to its tonic effect and flavor properties, which are mainly exerted by polysaccharides [11,32]. However, the attention to Polygonati Rhizome polysaccharides is still insufficient. This review aimed at providing updated and comprehensive information on Polygonati Rhizoma polysaccharides, hoping to broaden its usage as functional food.

## 2. Polysaccharide Phytosynthesis

Understanding the molecular mechanism of polysaccharide biosynthesis at the molecular level will be of benefit for modulating yield and properties of Polygonati Rhizoma polysaccharides in large-scale production. Polygonati Rhizoma polysaccharides are often referred to as *P. sibiricum* polysaccharide (PSP), *P. cyrtonema* polysaccharide (PCP), and *P. kingianum* polysaccharide (PKP) [33].

PSP content was positively correlated with the expression patterns of β-fructofuranosidase, fructokinase, mannose-1-phosphate guanylyltransferase, and UDP-glucose 6-dehydrogenase, but negatively correlated with the expression of hexokinase [34]. Most differential expression of genes in different parts of *P. sibiricum* rhizomes was assigned to the “starch and sucrose metabolism” pathway [35]. During the process of PSP biosynthesis, sucrose was converted to D-Glu-6-phosphate (D-Glu-6P), D-Fru, and D-Glu; then, D-Glu-6P was converted to α-D-Glu-1P by phosphoglucomutase or D-Fru-6P by Glu-6P isomerase isomerization [35]. In addition, UDP-glucose-6-dehydrogenase and UDP-glucuronic acid (GlcA) 4-epimerase accomplished the conversion for UDP-Glu, UDP-GlcA, and UDP-galacturonic acid (GalA). Finally, these activated sugar units were assembled into growing polysaccharide chains by corresponding glycosyltransferases [35].

The content of PKP was negatively correlated with fibrous root number, stem diameter, leaf width, leaf length, and the fresh weight of the above ground parts [36]. Of note, polysaccharide accumulation was greatly influenced by growth age. Among 2–5-year-old *P. kingianum*, polysaccharide content was the highest in 4-year-old plants by seed reproduction [36].

Based on transcriptome sequencing, 89 unigenes encoding enzymes associated with polysaccharide biosynthesis were found in *P. cyrtonema* [37]. The content of PCP was positively associated with expression levels of mannose-6-phosphate isomerase, Mevalonate kinase, 4-hydroxy-3-methylbut-2-enyl diphosphate reductase, UDP-apiose/xylose synthase, GDP-L-fucose synthase, hydroxymethylglutaryl CoA synthase, (E)-4-hydroxy-3-methylbut-2-enyl-diphosphate synthase, 2-C-methyl-D-erythritol 2,4-cyclodiphosphate synthase, and farnesyl diphosphate synthase [37]. UDP glycosyltransferases and transcription factors involved in polysaccharide biosynthesis were also identified [38].

## 3. Chemistry of Polygonati Rhizoma Polysaccharides

### 3.1. Summary of Extraction and Purification

Traditionally, Polygonati Rhizoma is pretreated with hot water to obtain crude polysaccharides, then subjected to ethanol precipitation to remove inactive substances and proteins [39]. Extraction efficiency could be affected by several factors, including temperature, time, extraction frequency, and solvent. The temperature, time, and liquid/solid ratio range within 80–100 °C, 2–3 h, and 1:(10–30) g/mL, respectively [30]. Meanwhile, Polygonati Rhizoma polysaccharides are often purified using DEAE-Cellulose52, DEAE-Sepharose Fast Flow ion-exchange chromatography, Sephadex G-75 gel filtration chromatography, and Sephacryl G-150 column chromatography [40,41]. In addition, enzymatic hydrolysis extraction, ultrasonic crushing and extraction, and microwave-assisted extraction, are also used for extraction of Polygonati Rhizoma polysaccharide [36]. Dilute lye can improve yield through destroying the cell wall and facilitating release of Polygonati Rhizoma polysaccharides from cell [36].

A high diversity of existing PSPs was reported in different research, which might be due to maturity, geographic location, environmental circumstances, extraction methods, and analytical procedures [30]. PSP was also extracted with CO_2_-triggered switchable hydrophilicity solvents containing amines and water, and a yield of 399.2 mg/g was obtained at a solid-liquid ratio of 1:20, extraction temperature of 50 °C, ultrasonic power of 500 W, and extraction time of 60 min [42]. No large-scale or industrialization extraction and purification methods of Polygonati Rhizoma polysaccharides have been reported. Thus, advanced extraction technology could be used to extract Polygonati Rhizoma polysaccharides, such as aqueous two-phase extraction [43,44], subcritical water extraction [45,46], pulsed electric field-assisted extraction [47,48], and membrane separation technology [49].

### 3.2. Structure of Polygonati Rhizoma Polysaccharides

Structures of Polygonati Rhizoma polysaccharides were often established by acid hydrolysis, Fourier transform infrared spectroscopy, nuclear magnetic resonance (NMR) spectroscopic analyses, high-resolution mass spectrometry, ultra-high-performance liquid chromatography-quadrupole trap tandem mass spectrometry (UHPLC-MS/MS), high-performance gel permeation chromatography, high-performance liquid chromatography (HPLC)-fluorescence detection with pre-column derivatization, acid hydrolysis combined with high-performance anion exchange chromatography with pulsed amperometric detection, methylation analysis combined with gas chromatography-mass spectrometry (GS-MS), as well as chemical evidence [50,51,52,53,54].

Polygonati Rhizoma polysaccharides and oligosaccharides could be decomposed into monosaccharides during the repeated steaming process [55]. Monosaccharide composition differed after steaming: the content of Gal and β-1,4-mannopyranose increased and the content of Glu and mannose (Man) decreased, while glycosidic linkage β-1,4-galactopyranose appeared [17]. The FTIR spectra showed that Polygonati Rhizoma polysaccharides possessed different degrees of esterification [56]. The microwave-assisted degradation approach could lower the molecular weight of PSP, but greatly improve bioactivity, such as antioxidant activity [57]. PSP, PCP, and PKP all had triple-helical structures with β-D-fructofuranosyl (Fruf), α-D-glucopyranose (Glcp, α-D-galactopyranose sugar residues, and an *O*-acetyl group [58].

There are five types of PSP, i.e., pectin [59,60,61], fructan [29], glucogalactomannan [30], arabinogalactan-type polysaccharides [62], and glucomannans [63]. Crude PSP contains carbohydrates (85.1–88.3%), proteins (4.51–11.9%), and uronic acid (1.79–7.47%) [36]. Methanolysis combined with the TFA hydrolysis method could be used for pectin extraction [60,61]. PSP contains different percentage compositions of Man (62.3–76.3%), Glu (15.2–20.3%), galactose (Gal) (4.35–15.3%), and arabinose (Ara) (4.00–7.65%), and small amounts of Fru, rhamnose (Rha), Xyl, GalA, and GlcA, as well as branched homogalactan and galactomannans (Table 1) [36]. Another report showed that crude PSP possessed high contents of Glu (15.1%), Gal (29.6%), and Man (36.1%) [64]. PSP0-PSP9, extracted from each cycle of steaming-drying, varied in molecular weights, but had similar backbones and chemical groups [65]. However, both biological and chemical variance of PSPs revealed considerable segregation of PSP0, PSP1- PSP4 and PSP5-PSP9 [65].

PCP mainly contained Glu, Man, Rha, Gal, ribose (Rib), and Ara [66,67]. PCP was formed by a branched fructan core with (2→6)-linked-*D*-Fruf residues every three (2→1)-linked-*D*-Fruf residues, and an average degree of polymerization of 28 [25]. PCP-1 possessed a (2→1)-linked β-*D*-Fruf backbone, as well as (2→6)-linked β-*D*-Fruf side chains with an internal α-*D*-Glc*p* in neokestose form [68]. PCP0-PCP5 (10^5^-10^7^ Da), extracted consecutively and steamed for 0–5 times, showed irregularly spherical conformation in aqueous solution [17,69]. The process of steaming led to significant structural changes of PCP, including molecular degradation, aggregation, and depolymerization [16]. The molecular weights of PCP changed during repeated steaming, first increasing with steaming and then decreasing with further steaming [17]. *P. cyrtonema* fructan contained a (2→6) linked β-*D*-Fruf residue backbone along with an internal α-D-Glc*p* residue and two (2→1) linked β-D-Fruf residue branches [70]. *P. cyrtonema* galactan was (1→4)-β-*D*-galactan branched with a single β-*D*-Gal at the C-6 at about every nine residues in the main chain [70].

PKP mainly contained Fru, Glu, Gal, Man, Xyl, Ara, Man, and β1,2-link Glu [71].

## 4. Applications for Health-Promoting Activities

Polygonati Rhizoma polysaccharides, Polygonati Rhizoma water extracts rich in polysaccharides, and herbal medicine formulation containing Polygonati Rhizoma polysaccharides all showed typical antioxidant, anti-aging, anti-fatigue, metabolic regulation, immunomodulatory, anti-inflammatory, anti-diabetic, antiatherosclerotic, hypolipidemic, anti-osteoporosis, and anti-cancer activities through a synergy mechanism [72].

### 4.1. Antioxidant and Anti-Aging Activities

Aging, a progressive decline of physiological functions in human life, is a major risk for various types of chronic diseases [73,74]. As the global elderly population will reach more than 2.1 billion by 2050, effectively alleviating aging is of great significance [75,76]. The number of diseases associated with aging increases gradually on a global scale [77,78]. In particular, aging is generally characterized by the imbalance between oxidative stress-induced damage and an organism’s antioxidant defenses [79,80,81]. Antioxidants, abundant in fruits, vegetables and herbs, can effectively resist oxidative stress [82,83]. As of now, there is no therapy or drugs able to cure aging-related diseases. Nutritional intervention might be an effective strategy to promote healthy aging and improve quality of life [84,85].

Polysaccharides of Polygonati Rhizoma exerted strong free radical-scavenging abilities, metal chelating activity, and significant reducing capacity [40,58]. PCP showed higher DPPH radical scavenging ability (IC_50_ = 2.04 mg/mL) than PSP (IC_50_ = 3.07 mg/mL) and PKP (IC_50_ = 4.10 mg/mL), while PSP exhibited higher ABTS radical-scavenging abilities (IC_50_ = 0.68 mg/mL) than PCP (IC_50_ = 0.85 mg/mL) and PKP (IC_50_ = 1.61mg/mL) [58]. At a concentration of 5.0 mg/mL, the hydroxyl radical-scavenging abilities of PCP, PSP, and PKP were 88.82%, 77.18%, and 72.75%, respectively [58]. As the steaming process progressed, the radical scavenging activity of PSPs increased gradually [65]. In heart-aging mice, PSP could decrease reactive oxygen species (ROS) and malondialdehyde (MDA), increase the level of superoxide dismutase (SOD), as well as inhibit DNA damages and lipid peroxidation through reducing expression of 8-hydroxydeoxyguanosine and 4-hydroxy-2-nonenal [86]. In atherosclerosis male rabbits, PSP protected endothelial cells from injury and apoptosis induced by H_2_O_2_ and lipopolysaccharide (LPS), and reduced the intimal foam cell number [87]. In HT-22 cells, PSP attenuated LPS-induced production of ROS [88]. PCPS (4.24 × 10^4^ Da) showed strong effects in preventing oxidative damage through activating erythroid 2-related factor 2 (Nrf2)/HO-1 antioxidant signaling [89]. PCP could reduce oxidative stress-induced ROS accumulation and alleviate ferroptosis via activating NRF2/HO-1 signaling pathway, thus attenuating neuronal-regulated cell death in microglia [90].

Polygonati Rhizoma polysaccharides could significantly reduce the MDA content of skeletal muscle and serum, enhance the activity of SOD and glutathione peroxidase (GSH-Px), and decrease free radical activity [91]. During the aging process, serious mitochondrial DNA damage and repair genes activities were observed. Polygonati Rhizoma polysaccharides exert an anti-aging effect through improving energy metabolism of liver mitochondria, reducing the expression of DNA polymerase γ, and enhancing the activities of respiratory chain enzyme complexes [91]. In mouse brain cells, Polygonati Rhizoma polysaccharides showed obvious anti-aging effects through increasing Na^+^-K^+^-ATP and Ca^2+^-ATP activity via Ca^2+^ overload, as well as reduced the level of lipid peroxide, lipofuscin, and B-type monoamine oxidase [91]. In natural menopausal rats, Polygonati Rhizoma polysaccharides delayed senescence through enhancing antioxidant capacity and improving blood lipid metabolism [36]. In D-Gal-induced rats, PSP (100 mg/kg) showed antiaging activity through effectively improving learning and memory abilities, reversing pathological changes of kidneys, down-regulating expression of the *FOXO3a* gene in renal tissue, regulating Klotho-fibroblast growth factor-23 endocrine axis, alleviating oxidative stress, and balancing calcium and phosphorus metabolism [92,93]. Collectively, PSP might serve as a potential effective constituent for anti-aging therapy.

### 4.2. Immunomodulatory Effects

Immunosuppression, a state of temporary or permanent immunity dysfunction, could make an organism more sensitive to pathogens. The search for effective methods to prevent and treat immunosuppressive diseases is vital. Natural products, such as herbal medicines rich in antioxidants, exert immunomodulatory effects towards chronic diseases through stimulating the immune system [94,95]. Natural immune modulators can effectively defeat disorders through up- or down-regulating immune response without undesired adverse effects [96,97]. Molecules, participating in immune activation, can induce immune responses against infectious diseases [98]. Polygonati Rhizoma possesses immune enhancement activities mainly through activating the immune system, improving growth and activity of immune cells, and promoting synthesis of antibodies [2].

**Figure 2:**
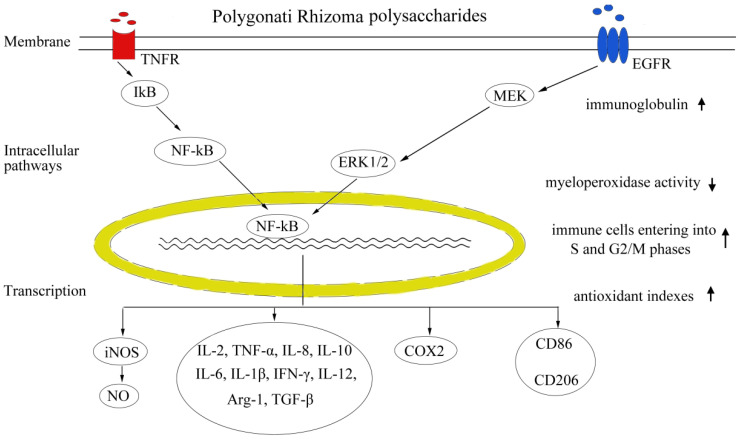
Schematic illustrating of immunomodulatory effects of Polygonati Rhizoma.

The immunomodulatory effect of polysaccharides from Polygonati Rhizoma is illustrated in Figure 2. In cyclophosphamide (CY)-induced immunosuppressed-mice, PSP can improve immunosuppression through recovering body mass, accelerating the recovery of spleen and thymus indexes, enhancing immunocyte proliferation responses (T cell, B cell, and splenocytes), elevating peritoneal macrophage phagocytosis, increasing blood erythrocyte counts, elevating CD4+/CD8+ ratio, accelerating the recovery of natural killer cell activity, and dose-dependently restoring the levels of serum immune factors, including interleukin (IL)-2, tumor necrosis factor (TNF)-α, IL-8, and IL-10 [39,99]. Polygonati Rhizoma polysaccharides can also promote the formation of hemolysin involved in humoral immune function, as well as improve phagocytic activity of peritoneal macrophages [36]. *P. cyrtonema* fructooligosaccharide, graminan-type fructan with a degree of polymerization of 5–10, could significantly reduce the level of pro-inflammatory cytokines (TNF-α, IL-1β) in serum, increase mice survival rate from 12.5% to 54%, and reduce inflammatory monocyte accumulation in lung tissue of peritonitis-induced mice [100]. In CY-induced immunosuppressed chickens (481-day-old), PSP showed protective effects via accelerating the recovery of relative weights of immune organs, stimulating immunoglobulin and antioxidant indexes in serum, improving the proliferation of peripheral blood T lymphocytes, promoting immune organs cells to enter into S and G2/M phases, upregulating the expression of immune factors (IL-2, IL-6 and interferon-γ), and inhibiting the apoptosis in spleen, thymus, and bursa of Fabricius [101]. Moreover, PSP also enhanced growth performance, as daily weight gain and serum protein production were elevated, while feed conversion ratios were decreased [101].

In RAW264.7 macrophages, Polygonati Rhizoma polysaccharides (100-400 μg/mL) regulated polarization through increasing secretion levels of pro-inflammatory cytokines (TNF-α, IL-12, IL-1β, and NO); reducing the levels of IL-10, arginase-1 (Arg-1), and TGF-β; elevating M1 characteristic surface molecule CD86; and reducing M2 surface molecule CD206 expression without any cytotoxic effects [58]. Particularly, M1-polarized macrophages could mediate host defense against the microbial infections and tumors, while M2 macrophages, featuring CD206, IL-10, Arg-1, and TGF-β, were involved in immune tolerance and tumor progression [58]. Furthermore, PSP-induced dendritic-like morphological changes caused IkB-α degradation, prompted NF-kB p65 translocation into the nucleus, and increased the production of immune-associated factors including NO, TNF-α, inducible nitric oxide synthase (iNOS), COX-2, NF-kB, phosphorylated p38 MAPK, and IL-6 [102]. PCP-1 exhibited immune-stimulating activity on cell viability and IL-6 production in RAW 264.7 macrophages [28]. CTAB-modified PSP-Cubs could enhance the proliferation of splenic lymphocytes [103].

Compared with raw rhizome, PCP steamed for 2–4 h had higher immunological activities [16]. Long steaming time (6–12 h) could exert negative impacts on the immunological activities of PCP [16]. PSPs (PSP1, PSP2, PSP3 and PSP4) with different monosaccharide composition and chemical structure exerted different abilities to activate phagocytic activity in vitro [41]. In particular, PSP3 possessed the best immunomodulatory function, showing great potential as an immunomodulator [41]. Steamed PCP significantly increased scavenging activity, while native PCP had the best immunostimulatory effect regarding NO production and phagocytosis [99].

### 4.3. Potential Antidiabetic/Antiobesity Effects

Diabetes mellitus (DM), including type 1 (T1DM) and type 2 (T2DM), is characterized by chronic metabolic disorder along with multiple organ failure [104,105]. In particular, T2DM is characterized by hyperglycemia due to a defect in insulin secretion of pancreatic β-cells and insulin resistance [106]. Polygonati Rhizoma has been used in the treatment of diabetes, hyperlipidemia, and related metabolic syndrome for centuries [107,108,109]. In particular, Polygonati Rhizoma polysaccharides alleviated hyperglycemia and reduced oxidative stress, and further delayed the progression of diabetic retinopathy and cataracts (Table 2).

In diabetic rats, PSP lowered the levels of fasting blood glucose (FBG) and glycated hemoglobin, improved clinical symptoms (polydipsia, polyphagia, polyuria and weight loss), delayed cataract progression, suppressed oxidative stress reaction, alleviated retinal vasculopathy, and elevated the levels of insulin and C-peptide in plasma [110,111]. Moreover, PSP can slow the progression of diabetic retinopathy and cataracts through alleviating hyperglycemia and reducing oxidative stress [111]. In NCI-H716 cells, Polygonati Rhizoma polysaccharides stimulated the production of glucagon-like peptide-1 [58]. In IR-3T3-L1 adipocytes, PSP alleviated inflammatory cytokines (IL-1β, IL-6, and TNF-α) and promoted Glu uptake via promoting Nrf2 expression [110]. PKPs-1 exhibited obvious anti-hyperglycemic activity through improving insulin tolerance and affecting metabolism of serum lipids [71]. Moreover, PKPs-1 increased expression of insulin receptor substrate-1, phosphoinositide 3-kinase (PI3K), and serine/threonine kinase (AKT), as the PI3K/AKT signaling pathway was involved in the regulation of Glu metabolism [71]. In T2DM rats, oral administration of PKP increased the content of fasting insulin and lowered the levels of FBG [109].

The water extract of Polygonati Rhizoma and Codonopsis Radix (PRCR), mainly the polysaccharide fraction, showed effective hypoglycemic effects through upregulation of the IRS1/PI3K/AKT signaling pathway and inhibition of IRS1 phosphorylation in the T2DM mouse model treated with PRCR extracts, as levels of total cholesterol, and triacylglycerol, alanine aminotransferase and aspartate aminotransferase were significantly reduced compared with the non-treated model group mice [112]. Polysaccharides and water extract of *P. kingianum* rhizome could alter the abundance of gut microbes and miRNAs expression, which further regulate lipid metabolism in HFD-rats [113]. In particular, the miR-484-Bacteroides/Roseburia axis acted as an important bridge hub, connecting the entire miRNA-gut microbiota network [113].

### 4.4. Bone Homeostasis Benefits

Osteoporosis, a common systemic skeletal disease, could affect 40% of Chinese women and cause more than 2 million osteoporotic fractures every year [114,115]. Osteoporosis is characterized by low bone mass, increased bone fragility, degeneration of microstructure, and susceptibility to fractures [50]. In particular, the occurrence of osteoporosis is increasing as populations age, and estrogen deficiency is speculated to be the most common cause of osteoporosis [50].

PSP showed protective effects on ovariectomy-induced bone loss in rats through increasing bone mineral density, enhancing expression of basic fibroblast growth factor, lowering expression of bone gla protein and tartarate-resistant acid phosphatase, as well as decreasing levels of sera TNF-α and bone-specific alkaline phosphatase [19]. In mice, PSP could promote osteogenic differentiation of bone marrow stromal cells through the increasing nuclear accumulation of β-catenin and elevating expression of osteoblast-related genes [116]. Moreover, PSP inhibited the receptor activator of NF-κB ligand (RANKL)-induced osteoclastogenesis, and exerted prophylatic protection against LPS-induced osteolysis in mice [116]. PSP inhibited osteoporosis through promoting osteoblast formation and blocking osteoclastogenesis via the Wnt/β-catenin signalling pathway [116].

In bone mesenchymal stem cells, PSP can promote osteoblastic differentiation through increasing nuclear accumulation of β-catenin via the ERK/glycogen synthase kinase 3β (GSK-3β)/β-catenin signaling pathway, as PSP upregulated nuclear β-catenin and reduced the level of GSK-3/β [117]. Crude PSP showed osteogenic activity through promoting the differentiation and mineralization of MC3T3-E1 cells in vitro [50]. In bone-marrow-derived mice macrophages, PSP inhibited osteoclastogenesis through the Hippo signaling pathway based on miR-1224, as the expression level of the target gene Limd1 was significantly increased [118].

### 4.5. Antimicrobial Activity

PSP could effectively promote the biomass, biofilm, and acetic acid production in *Lactobacillus faecis*, a specific probiotic in the intestinal tract [119]. Particularly, PSP promoted the quorum sensing system of *L. faecis* through enhancing the transcription of oppA and expression of oppD protein [119]. The proliferation of beneficial microbiotas, containing Parabacteroides and Bifidobacterium, was positively associated with PSP treatment [120]. PSP from steamed rhizome could significantly convert fatty acids into short-chain fatty acids (like acetic acid and propionic acid), and long-chain fatty acids (like *cis*, *cis*, *cis*-9,12,15-linolenic acid, *cis*-6-octadecenoic acid, and *cis*-9-octadecenoic acid) [120]. Furthermore, PSP also regulated the production and metabolism of short-chain fatty acids of *L. faecis* via upregulating the expression of *ldh* and *metE* gene and ADH2 protein, and downregulated the expression of the mvK gene [119]. Similarly, fructan and galactan extracted from *P. cyrtonema rhizome* possess prebiotic activity, which could remarkably promote the growth of Bifidobacterium and Lactobacillus strains [70].

PSP decreased the abundance of harmful microbiota *Shigella* [120]. PSP could inhibit the growth of *Escherichia coli*, *Bacillus subtilis*, and *Staphylococcus aureus* with the minimum inhibitory concentration (MIC) of 1.23 mg/mL, 0.98 mg/mL, and 1.31 mg/mL [121], respectively. The hydrolyzed PCP fragment B3, containing 1-kestose and the neokestose series of oligosaccharides without branches, showed antiherpetic activity against herpes simplex virus type 2 (HSV-2) in vero cell culture [122]. Compared with PCP and sulfonylated derivative, the phorphorylated derivative or sulfated derivative exhibited higher inhibitory activity against HSV, inferring that function groups were important for the antiherpetic activity [123]. In T2DM rats, oral administration with PKP could improve intestinal microecology through decreasing the abundances of *Bacteroidetes* and *Proteobacteria*, but increasing that of *Firmicutes* [109].

### 4.6. Anti-Fatigue Activities and Anti-Depression Benefits

Fatigue, a failure to maintain the required or expected power output in people with stress, is generally divided into physical and mental fatigue [124,125]. Depression has been listed as a particularly impactful disability [126]. Production of ROS and activation of calpain system and NOD-like receptor protein 3 (NLRP3) inflammasome are tightly related to depression [88]. Modulation of antioxidant enzymes activity by Polygonati Rhizoma polysaccharides might also effectively alleviate the exercise-induced oxidative stress and body fatigue.

In male C57BL/6 mice with fatigue, PSP could prevent depression-like behaviors, and synaptic and neuronal damage through reducing ROS/HPA axis hyperfunction and inflammatory response [127]. Moreover, PSP administration has greatly promoted the hippocampal expression of p-Akt, the mammalian target of rapamycin (mTOR), GluA1, and GluA2; reduced the expression of GluN2A, caspase-3, and GluN2B; and prevented the loss of granular cells in the DG region [127]. Polygonati Rhizoma polysaccharides might also reduce fatigue through decreasing blood lactate and serum urea nitrogen, as well as increasing liver and muscle glycogen [128,129]. In mice, PCP exerted antidepressant effects via regulating the oxidative stress-calpain-1-NLRP3 signaling axis, as the expression of calpain-1, NLRP3, apoptosis-associated speck-like protein, caspase-1, cleaved-caspase-1, ionized calcium binding adapter molecule 1, phosphorylation of extracellular signal-regulated kinase, NF-κB, and glial fibrillary acidic protein were reduced, while expression of calpastatin, phosphatase and tensin homolog, suprachiasmatic nucleus circadian oscillatory protein, and Nrf2 were increased [88].

### 4.7. Other Health-Promoting Activities

In Balb/c female mice bearing triple negative breast cancer (TNBC), polysaccharide-rich extract from *P. sibiricum* (PREPS) could protect hematopoiesis through inhibiting hematopoietic cell expansion in spleen, as well as markedly increase hematopoietic stem and progenitor cells and common lymphoid progenitors in the bone marrow [130]. PREPS may show long-lasting anti-tumor effects in assisting TNBC therapies through sustaining hematopoiesis and lymphoid regeneration in bone marrow [130]. In a HepG2 cell, the water-soluble PSP (38.65 kDa) showed concentration-dependent anticancer effects through arresting the cell cycle at the G1 phase, decreasing mitochondrial membrane potential, damaging the nucleus, and inducing cell apoptosis via increasing the activity of caspase-9 and caspase-3 [131].

In male Sprague-Dawley rats, PSP could prevent acute heart failure induced by adriamycin through exerting anti-oxidative activity, anti-inflammatory activity, and inhibition of cardiac myocyte apoptosis [132]. In rats with acute heart failure, PSP (400 mg/kg, 5 days) could increase heart rate, ±dp/dt_max_, myocardial Na^+^-K^+^-ATPase, Ca^2+^-Mg^2+^-ATPase, succinate dehydrogenase levels, serum superoxide dismutase level, left ventricular systolic pressure, as well as myocardial Bcl-2 and Caspase-3 protein expression levels [132]. Meanwhile, PSP significantly decreased the expression levels of left ventricular end diastolic pressure, serum biochemical indexe levels (cardiac Troponin-I, creatine kinase-MB isoform, TNF-α, IL-6, MDA, and NO), as well as myocardial Bax and cleaved Caspase-3 protein [132].

*P. cyrtonema* fructooligosaccharide could alleviate lung injury via ameliorating the damage of pulmonary cellular architecture in lung tissue of peritonitis-induced mice [100]. In mice with lung injury, raw and honey-processed PCP could increase levels of SOD, inhibit pulmonary inflammation through the NF-κB pathway, and reduce the occurrence of pulmonary oxidative stress via the AMPK-Nrf2 pathway [133]. The protective effect in lung injury may be tightly related to the antioxidant and anti-inflammatory activities of PCP [134,135].

Alzheimer’s disease (AD), a typical age-related dementia and progressive neurodegenerative disorder, is characterized by degeneration and loss of brain neurons, as well as memory loss [136,137]. Particularly, beta-amyloid (Aβ) peptide and its aggregates contribute much to the pathogenesis and progression of AD [136]. In the male mice model of dementia, PSP could greatly improve learning and memory by reducing the damaging effects of cerebral ischemia and anti-oxidation, as SOD and GSH-Px activity were elevated [138]. The protective effects of PSP against Aβ_25–35_-induced neurotoxicity in PC12 cells were mainly through attenuating cell death, elevating Bax/Bcl-2 ratio, inhibiting mitochondrial dysfunction and cytochrome C release into the cytosol, inhibiting caspase-3 activation, and enhancing the pro-survival PI3K/Akt signaling pathway as the level of phosphorylated Akt was elevated [136]. In particular, the regulation of mitochondrial permeability and the release of cytochrome c from the mitochondria to the cytosol are pivotal in the apoptotic repertoire, which are tightly controlled by the Bcl-2 protein family [136]. In D-gal-injured mice, PSP could significantly ameliorate synaptic injury, prevent cell death, increase anti-oxidative stress-related protein expression, and decrease inflammation-related protein expression [139].

In BALB/c mice, PSP potentially plays a protective role towards septic acute liver injury by inhibiting pyroptosis via NLRP3/GSDMD signals [140]. In particular, PSP treatment remarkably alleviated liver histopathologic damage, lowered the activity of neutrophil infiltration marker MPO in liver, decreased levels of liver function indexes and inflammatory cytokines (TNF-α and IL-6), decreased the expression of pyroptosis-related cytokines (IL-18 and IL-1β) in serum, restrained excessive pyroptosis, and reduced the 48 h mortality rate [140]. Moreover, water extracts of *P. kingianum* rhizome served as useful mitochondrial regulators/nutrients in remedying mitochondrial dysfunction and alleviating non-alcoholic fatty liver disease in rats [141].

In human kidney cells, PKP and PKAE alleviated uranium-induced cytotoxicity by regulating mitochondria-mediated apoptosis and the GSK-3β/Fyn/Nrf2 pathway: mitochondrial membrane potential and ATP level were increased, while ROS decreased [142].

In mice with blood deficiency syndrome, PSP significantly increased the peripheral blood cells, restored splenic trabecular structure, and reversed hematopoietic cytokines to normal levels through regulating the expression of genes involved in hematopoiesis and immune regulation signaling pathways [143]. Specifically, the blood-enriching effects of PSP were exerted through regulating the JAK1-STAT1 pathway and elevating hematopoietic cytokines (erythropoietin, granulocyte colony stimulating factor, TNF-α and IL-6) [143].

## 5. Future Prospects

Polygonati Rhizoma possesses sweet fragrance and taste, and is well-known as a traditional medicinal herb and functional food with significant health-improving effects. These members of the *Liliaceae*, *P. kingianum*, *P. sibiricum*, and *P. cytomema,* are mainly distributed throughout the temperate regions of northern hemisphere, especially China [144,145]. Their anti-fatigue, anti-aging, immunomodulatory, metabolic regulatory, anti-inflammatory, neuroprotective, and anti-cancer effects might be exerted through synergy mechanism [146,147]. Among the abundant biological substances, polysaccharides are believed to be one of the most important active compounds of Polygonati Rhizoma. Furthermore, the water-soluble polysaccharides are suitable for long-term administration for various health-promoting effects.

In modern society, environment pollution and competition intensification lead to increasing pressure, which results in immune system decline, premature aging, cancer, and other sub-health phenomena. Maintaining health and slowing down aging have become people’s great goals. Served as an antioxidant, Polygonati Rhizoma polysaccharides can delay senescence, and be used as an immunostimulant agent for protection against immunosuppression. Polygonati Rhizoma polysaccharides are also used to alleviate dryness, improve anti-osteoporotic fractures, promote the secretion of fluids, enhance viability of mesenchymal stem cells in bones, and quench thirst [34]. Moreover, Polygonati Rhizoma water extracts rich in polysaccharides and herbal medicine formulations containing Polygonati Rhizoma polysaccharides also show great potential in health-promoting activities in the health care and pharmaceutical industries [148,149,150].

The recent development of therapeutic agents using PSP to enhance bone health and prevention of osteoporosis is promising. NF-kB and p38 MAPK pathways might contribute much to the immunological activity of Polygonatum Rhizoma polysaccharides. Research on the chemical composition and molecular structure of Polygonati Rhizoma polysaccharides is still incomplete. The large-scale isolation and purification of the bioactive components from *P. kingianum*, *P. sibiricum*, and *P. cyrtonema* is not feasible due to the extremely low propagation rate. Therefore, more studies are greatly needed to discover their mechanisms of health-promoting activities in vivo and in vitro for their safe application in human health care.

## Figures and Tables

**Table 1:** Structure comparison among PCP, PKP, and PSP polysaccharides.

Type	Molecular Weight	Composition	Reference
PSP	(2.2–4400) × 10^3^ Da	Man, Glu Gal, Ara, Fru, Rha, Xyl, GalA, GlcA, homogalactan, and galactomannans	[36,58]
PCP	(8.5–42,400) × 10^3^ Da	Glu, Man, Rha, Gal, Rib, Ara, Fruf, Glcp	[66,67]
PKP	8.7 × 10^3^ Da	Fru, Glu, Gal, Man, Xyl, Ara, Man, and β1,2-link Glc	[58]

**Table 2:** Metabolic regulation mechanism of Polygonati Rhizoma polysaccharides.

Model	Active Components	Dose	Putative Mechanism	References
NCI-H716 cells	Polygonatum polysaccharides	25–100 μg/mL for 2h	stimulate GLP-1 production	[58]
HepG2 cells	PKPs-1	0.78–100 mg/L for 24 h	upregulate the levels of Glu utilization efficiency	[66]
STZ-induced diabetic mice	PKPs-1	1190 mg/kg once daily for 15 consecutive days	improve insulin tolerance; affect metabolism of serum lipids; activate PI3K/AKT signaling pathway; increase expression of IRS-1, PI3K, and AKT	[66]
STZ-induced diabetic SD rats	PSP	200–800 mg/kg·d for 12 weeks	lower levels of FBG and glycated hemoglobin; improve polydipsia, polyphagia, polyuria and weight loss; delay cataract progression; suppress oxidative stress reaction; alleviate retinal vasculopathy; elevate levels of insulin and C-peptide in plasma; inhibit formation of advanced glycosylation end products	[108]
T2DM rats	PKP	0.1 g/kg for 56 days	increase the content of fasting insulin and lowered the levels of FBG	[109]
IR-3T3-L1 adipocytes	PSP	50–250 µg/mL	alleviate inflammatory cytokines; promoting Nrf2 expression	[110]

## Data Availability

Not applicable.

## Abbreviations

Aβ, beta-amyloid; AD, Alzheimer’s disease; AKT, serine/threonine kinase; Ara, arabinose; Arg-1, arginase-1; CY, cyclophosphamide; DM, Diabetes mellitus; FBG, fasting blood glucose; Fru, fructose; Fruf, fructofuranosyl; Gal, galactose; GalA, galacturonic acid; GlcA, glucuronic acid; Glcp, glucopyranose; Glu, glucose; GSH-Px, glutathione peroxidase; GSK-3β, glycogen synthase kinase 3β; NLRP3, NOD-like receptor protein 3; HSV-2, herpes simplex virus type 2; IL, interleukin; iNOS, inducible nitric oxide synthase; LPS, lipopolysaccharide; Man, mannose; MDA, malondialdehyde; MIC, minimum inhibitory concentration; mTOR, mammalian target of rapamycin; Nrf2, nuclear factor erythroid 2-related factor 2; PCP, Polysaccharides from *P. cyrtonema;* PI3K, phosphoinositide 3-kinase; PSP, Polysaccharides from *P. sibiricum;* PKP, Polysaccharides from *P. kingianum;* PREPS, polysaccharide-rich extract from *P. sibiricum;* ROS, reactive oxygen species; Rha, rhamnose; SOD, superoxide dismutase; T1DM, type 1 diabetes mellitus; T2DM, type 2 diabetes mellitus; TNBC, triple negative breast cancer; TNF, Tumor necrosis factor; Xyl, xylose
